# The Use of a Humanized NSG-β2m^−/−^ Model for Investigation of Immune and Anti-tumor Effects Mediated by the Bifunctional Immunotherapeutic Bintrafusp Alfa

**DOI:** 10.3389/fonc.2020.00549

**Published:** 2020-04-21

**Authors:** Y. Maurice Morillon, Claire Smalley Rumfield, Samuel T. Pellom, Ariana Sabzevari, Nicholas T. Roller, Lucas A. Horn, Caroline Jochems, Claudia Palena, John W. Greiner, Jeffrey Schlom

**Affiliations:** Laboratory of Tumor Immunology and Biology, Center for Cancer Research, National Cancer Institute, National Institutes of Health, Bethesda, MD, United States

**Keywords:** humanized mouse, NSG-β2m^−/−^, bintrafusp alfa, immunotherapy, TGF-β

## Abstract

The lack of serial biopsies in patients with a range of carcinomas has been one obstacle in our understanding of the mechanism of action of immuno-oncology agents as well as the elucidation of mechanisms of resistance to these novel therapeutics. While much information can be obtained from studies conducted with syngeneic mouse models, these models have limitations, including that both tumor and immune cells being targeted are murine and that many of the immuno-oncology agents being evaluated are human proteins, and thus multiple administrations are hampered by host xenogeneic responses. Some of these limitations are being overcome by the use of humanized mouse models where human peripheral blood mononuclear cells (PBMC) are engrafted into immunosuppressed mouse strains. Bintrafusp alfa (M7824) is an innovative first-in-class bifunctional fusion protein composed of the extracellular domain of the TGF-βRII to function as a TGF-β “trap” fused to a human IgG1 antibody blocking PD-L1. A phase I clinical trial of bintrafusp alfa showed promising anti-tumor efficacy in heavily pretreated advanced solid tumors, and multiple clinical studies are currently ongoing. There is still much to learn regarding the mechanism of action of bintrafusp alfa, including its effects on both human immune cells in the periphery and in the tumor microenvironment (TME), and any temporal effects upon multiple administrations. By using the NSG-β2m^−/−^ mouse strain humanized with PBMC, we demonstrate here for the first time: (a) the effects of bintrafusp alfa administration on human immune cells in the periphery vs. the TME using three different human xenograft models; (b) temporal effects upon multiple administrations of bintrafusp alfa; (c) phenotypic changes induced in the TME, and (d) variations observed in the use of multiple different PBMC donors. Also discussed are the similarities and differences in the data thus far obtained employing murine syngeneic models, from clinical trials, and in the use of this humanized mouse model. The results described here may guide the future use of this agent or similar immunotherapy agents as monotherapies or in combination therapy studies.

## Introduction

The success of immuno-oncology in the management of subsets of patients with certain tumor types is well-established. However, with only ~15–25% of objective responses (OR) in many carcinoma types, more needs to be learned about the mechanisms involved in these approaches. The lack of serial biopsies in patients with a range of carcinomas has been one obstacle in our understanding of immuno-oncology agents. While much information has been gleaned from syngeneic mouse models, these models have limitations: they are composed of both murine immune and tumor cells. Moreover, many of the immuno-oncology agents being evaluated are human proteins, and thus multiple administrations are hampered by host xenogeneic responses. Investigation of immunotherapeutic agents in peripheral blood mononuclear cell (PBMC) humanized mice has primarily utilized NSG, NRG, and NOG mice. Studies have revealed anti-tumor efficacy and mechanistic insight into the human immune mediated anti-tumor response. The primary caveat to the PBMC humanized model is the rapid development of Graft vs. Host Disease (GvHD), the kinetics of which limits the duration of study and interpretation of results. Several variants of these parental mouse strains have eliminated murine MHC class I and/or II and exhibit delayed development of GvHD in the PBMC model (1–5). Due to their recent development, few immunotherapeutic studies have utilized these novel lines, and while limitations exist, delayed development of GvHD enables more complete characterization of immunotherapeutic modulation of the human anti-tumor response. One such variant is the NSG-β2m^−/−^ strain, which is utilized in the current study. This model represents a potential intermediate between syngeneic models and clinical studies. The NSG-β2m^−/−^ mouse lacks expression of beta-2 microglobulin (β2m), eliminating murine MHC class I and resulting in the delayed development of GvHD in PBMC humanized mice. This advantage was highlighted in a recent study which observed a significant survival advantage in NSG-β2m^−/−^ mice injected with human PBMC when compared to the parental NSG line (1). To our knowledge, this is the first study to utilize the NSG-β2m^−/−^ PBMC model in the investigation of immunotherapeutic modulation of a human anti-tumor response.

Bintrafusp alfa (M7824) is an innovative first-in-class bifunctional fusion protein composed of the extracellular domain of the TGF-βRII to function as a TGF-β “trap” fused to a human IgG1 antibody blocking PD-L1. Several prior studies have been carried out characterizing the biologic activity of bintrafusp alfa both *in vitro* and *in vivo*, employing both murine and human immune cells and tumor cells (6–10). Bintrafusp alfa was shown to be capable of mediating antibody-dependent cell-mediated cytotoxicity (ADCC) for a wide range of human carcinoma cells *in vitro* employing human natural killer (NK) cells as effectors (8). These studies also showed that the reduction of NK activation markers, and NK lytic activity of tumors, induced by TGF-β1 could be abrogated by bintrafusp alfa but not by anti-PD-L1. Bintrafusp alfa, but not anti-PD-L1, was also shown to reduce the immunosuppressive activity of human regulatory T cells (Tregs) on human CD4^+^ T-cell proliferation (8). Compared to anti-PD-L1, bintrafusp alfa was also shown (7) to increase the gene expression of molecules involved in T-cell trafficking in the tumor (e.g., CXCL11), TRAIL-mediated tumor cell lysis, and antigen-specific T-cell lysis of tumor cells. Prior studies (6) have also shown that TGF-β1 serves as a molecular link between human lung tumor cell mesenchymalization and elevated PD-L1 expression and that this mesenchymalization was effectively antagonized using bintrafusp alfa, but not by anti-PD-L1.

Two studies (9, 10) have reported the advantage in anti-tumor activity of bintrafusp alfa or a similar anti-PD-L1/TGF-βRII molecule over the use of a combination of anti-PD-L1 plus a TGF-β blocking agent. To better define the contribution of the anti-PD-L1 vs. the TGF-βRII components of bintrafusp alfa, a recent study (11) in murine models compared bintrafusp alfa to a bintrafusp alfa mutant devoid of its anti-PD-L1 binding site. The ability to block PD-L1 and sequester TGF-β was required for the anti-tumor efficacy of bintrafusp alfa, as TGF-β sequestration alone by the bintrafusp alfa mutant did not improve anti-tumor responses. Moreover, while the bintrafusp alfa mutant was able to decrease TGF-β1 levels in the plasma, it did not bind to TME cells expressing PD-L1 and, in contrast to bintrafusp alfa, did not decrease TGF-β-dependent signaling in the TME (11).

Bintrafusp alfa is currently being studied in clinical trials at multiple institutions. Human *in vitro* studies, and studies in syngeneic mouse models, involving bintrafusp alfa have been previously reported (6–9, 11). The phase I, open-label, dose-escalation, and dose-expansion clinical trial showed promising anti-tumor efficacy in heavily (12–15) pretreated advanced solid tumors. Bintrafusp alfa showed a safety profile similar to anti-PD-1/PD-L1 monotherapies (14, 15). Of the 19 patients enrolled in the initial study at the National Cancer Institute (15), one patient (cervical cancer) demonstrated a durable complete response, two patients (pancreatic, anal) had durable partial responses, and two patients (pancreatic, carcinoid) experienced prolonged stable disease. Expansion cohorts of the phase I study of bintrafusp alfa have also shown promising results. For example, among 80 second-line non-small cell lung cancer patients, there was an overall response rate (ORR) of 27.5% at the 1,200 mg dose; among PD-L1 high patients, a 71% ORR was observed at this dose (16). In other phase I expansion cohorts, early signs of clinical efficacy were also observed in Asian patients with recurrent gastric (17) or gastroesophageal junction (17) cancers and with pretreated biliary tract cancer (18).

Preliminary results from an ongoing phase I/II clinical trial (NCT02517398) of patients with human papilloma virus (HPV)-associated malignancies including cervical, anal, or head and neck squamous cell carcinoma are encouraging (14, 15). Of the 36 patients who received bintrafusp alfa, there was a clinical response rate of 38.9% with an acceptable safety profile (15). Prior studies with anti-PD-1/PD-L1 agents have demonstrated 15–25% response rates in this patient population. Also ongoing are multicenter Phase II studies of bintrafusp alfa in lung, biliary, and other cancers with promising results. However, much still needs to be learned in terms of the mechanism of action of bintrafusp alfa, including its effects on both human immune cells in the periphery and in the tumor microenvironment (TME), and any temporal effects upon multiple administrations.

In the studies reported here, we describe for the first time: (a) comparative effects of bintrafusp alfa administration on human immune cells in the periphery vs. the TME using three different human xenograft models; (b) temporal effects upon multiple administrations of bintrafusp alfa; (c) phenotypic changes induced in the TME, and (d) variations observed in the use of multiple different PBMC donors. Also discussed are the similarities and differences in the data thus far obtained employing murine syngeneic models, from clinical trials, and in the use of this humanized mouse model.

## Materials and Methods

### Mice

NSG-β2m^−/−^ mice were obtained from the Jackson Laboratory (Bar Harbor, ME) and bred and maintained in microisolator cages under SPF conditions in accordance with AAALAC guidelines. All animal studies were performed following approval from the NIH Intramural Animal Care and Use Committee.

### Cells

HTB-1 human transitional cell carcinoma, MDA-MB-231 triple negative breast adenocarcinoma, and SiHa HPV positive squamous cell carcinoma were obtained from ATCC and cultured according to the manufacturer's specifications. Healthy donor PBMCs were obtained from the NIH Blood Bank, processed, and stored as previously described (19).

### Murine PBMC Engraftment and Tumor Injection

5 × 10^6^ (HTB-1, MDA-MB-231) or 3 × 10^6^ (SiHa) cells were combined with Matrigel at a 1:1 ratio immediately prior to subcutaneous (s.c.) injection into the right flank of recipient animals in a total volume of 100 μl. Seven days post-tumor injection, PBMCs were thawed in a 37°C water bath, followed by three washes with RPMI containing 10% FBS, and one wash with PBS. 1 × 10^7^ PBMCs were injected intraperitoneally (i.p.) in a volume of 200 μl PBS. Tumors were measured twice weekly using calipers, and the tumor volume was calculated as: Volume = 0.5 × (width)^2^ × (length). Animals were weighed weekly with weight loss calculated as a percent change from the first measurement. Animals exhibiting a >15% loss in body weight were euthanized. Proteinuria was determined weekly using a drop of urine placed on Albustix (Siemens, Malvern, PA).

### Treatments

Bintrafusp alfa was constructed and produced by EMD Serono and provided under a Cooperative Research and Development Agreement (CRADA) with the NCI, NIH. Bintrafusp alfa was administered once weekly at a dose of 500 μg/injection delivered via the i.p. route starting seven days post-PBMC reconstitution.

### Fluorescent Labeling of Bintrafusp Alfa

An azide residue was added to bintrafusp alfa using the SiteClick Antibody Azido Modification Kit (Thermo Fisher Scientific, Waltham, MA), followed by fluorescent labeling using the Click-iT Alexa Fluor 647 sDIBO Alkyne for Antibody Labeling Kit (Thermo Fisher Scientific), according to the manufacturer's instructions. Labeled bintrafusp alfa or label alone was injected i.p. at a dose of either 5 or 50 μg into HTB-1 tumor bearing mice. Two weeks post-injection, animals were euthanized, with organs and tumors collected. Whole organ/tumor imaging was performed using the IVIS Lumina *in vivo* Imaging platform (Caliper Life Sciences, Almeda, CA).

### Flow Cytometry

Tumors and spleens were excised and homogenized via mechanical dissociation. Red blood cells were lysed using ACK buffer (Quality Biologicals Inc., Gaithersburg, MD) and single cell suspensions were prepared by filtering through a 40-micron nylon cell strainer. PBMCs were isolated from blood utilizing Lympholyte-M (Cedarlane Laboratories, Burlington, NC).

Cell suspensions were stained on ice with fluorescently conjugated antibodies diluted in FACS buffer. Dead cells were identified via Live/Dead fixable stain (ThermoFisher Scientific, Waltham, MA). When necessary, intracellular staining was performed using FoxP3/transcription factor kit (eBioscience, San Diego, CA), according to the manufacturer's instructions. Cells were enumerated utilizing AccuCheck Counting beads (ThermoFisher). T-cell IFNγ production was detected via *ex vivo* incubation at 37°C with Cell Stimulation Cocktail (eBioscience) diluted 1:500 in RPMI for 5 h with the addition of GolgiPlug (ThermoFisher) for the final 4.5 h of the culture.

Antibodies used for flow cytometry included: Human (h) CD3 (AF700), hCD4 (BV711), hCD8 (BV785), hIFNγ (PerCP-Cy5.5), hGzmB (FITC), hCD45 (AF647), hPD-L1 (PE-Cy7), murine CD45 (APC-Cy7), and either hPD1 (PE) or hNKG2D (PE). Antibodies used for flow cytometry were purchased from Biolegend (San Diego, CA). Cytometric data were obtained via a 4 laser Attune (ThermoFisher). Data were analyzed via FlowJo (FlowJo, LLC, Ashland, OR).

### Microscopy

Tumors were fixed with Z-Fix Aqueous buffered zinc formalin fixative (Anatech LTD, Battle Creek, MI). Tumors were embedded, sectioned, and stained by HistoTox Labs (Boulder, CO). Imaging was performed using an LSM 880 NLM Airyscan confocal microscope (Zeiss, Thornwood, NY).

### NanoString Analysis

#### RNA Isolation

Tumors were dissociated using either the Human Tumor Dissociation kit (Miltenyi Biotec, San Diego, CA) and gentleMACS dissociator (HTB-1), or mortar and pestle in RNeasy RLT buffer with β-mercaptoethanol (SiHa). Tumors were CD45 enriched using the human CD45 (TIL) Isolation kit (Miltenyi Biotec). RNA was isolated using the RNeasy mini kit (Qiagen).

#### NanoString Analysis With nSolver

Data from a NanoString PanCancer Immune Profiling Panel were imported into the Nanostring nSolver software. The geometric mean of the negative controls was calculated and subtracted from the counts of all samples. Positive control and housekeeping normalization was performed using the geometric mean of a set of positive control and housekeeping genes. Ratios and fold change estimations were built by comparing treatment to control samples.

#### Ingenuity Pathway Analysis (IPA)

Files containing grouped fold change and *p*-values were exported from nSolver and imported into the IPA software. Cutoffs for the analysis were a *p*-value of < 0.1 and a fold change of < −1.5 or >1.5. Pathways were filtered to include only those relevant to immune activity and whose z-score was not undefined.

#### Partek Genomics Suite

Normalized log2 counts for HTB-1 samples were imported into the Partek analysis software. An ANOVA was run comparing bintrafusp alfa against control samples. Normalized log2 counts of SiHa tumors were narrowed to those genes present on the HTB-1 gene list using Python 3.7 and Jupyter-Lab with the Pandas package. A hierarchical clustered heatmap was created from genes having a fold change of < −1.5 or >1.5 and a *p*-value of < 0.1. Fold change was standardized by shifting expression of genes across samples to a mean of zero with a standard deviation of one. Scaling from the HTB-1 analysis was used for both the HTB-1 and SiHa heatmaps.

#### Cytoscape

Genes from the top five identified IPA pathways were imported into the CytoScape STRING app. A confidence score cutoff of 0.70 with additional maximum interactions of zero was utilized. A network of interaction was created with unconnected nodes removed.

#### Immune Activation Score

Immune activation scoring was calculated as previously described (20). Briefly, the immune activation score was determined by taking the geometric mean of normalized counts for each of the following genes inserted into the following equation:

Score = $\sqrt[{2}]{(}\sqrt[{12}]{(}$CTSH × CD74 × HLA-DPB1 × HLA-DQB1 × HLA-DMB × HLA-DOB × HLA-DRB3 × HLA-DRB4 × HLA-DQA1 × HLA-DPA1 × HLA-DMA × HLA-DRA)) × ($\sqrt[{6}]{(}$ IFNG × CD4 × PDCD1 × CD274 × IL7R × CD3D)).

### Serum/Tumor TGF-β Detection

Serum/blood was collected via submandibular venipuncture into BD Microtainer SST collection tubes (BD Bioscience), followed by centrifugation and storage at −20°C. Tumors were mechanically dissociated in PBS and added at equal volume to 2X Cell Lysis Buffer (Item J, RayBiotech, Inc., Peachtree Corners, GA) with Protease Inhibitors included (RayBiotech, Inc.). Tumors were sonicated, lysates centrifuged at 10,000 RCF, and supernatants stored at −20°C.

Serum and tumor TGF-β were measured via ELISA (R&D Systems, Minneapolis, MN), according to the manufacturer's instructions. Endogenous active TGF-β was determined by omitting the acid activation step. Total protein concentration was determined via BCA assay (ThermoFisher).

### Statistical Analysis

GraphPad Prism 7 (GraphPad Prism Software, Inc., La Jolla, CA) was used to perform statistical analyses. Details of the appropriate analysis are found within each figure legend.

## Results

### PBMC Humanized NSG-β2m^-/-^ Model

**Figure 1:**
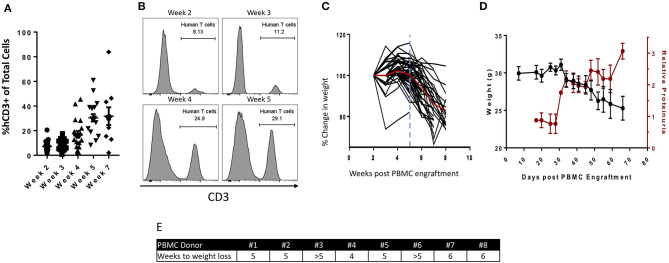
Characterization of the PBMC humanized NSG-β2m^−/−^ mouse model. 1 ×10^7^ human healthy donor PBMCs were injected i.p. into NSG-β2m^−/−^ mice at day 0. **(A)** Animals were bled weekly with circulating lymphocytes isolated and immunostained for human CD3. Graph depicts frequency of human CD3^+^ cells of total live circulating cells at weeks 2–7 post-PBMC engraftment (*n* = 19; week 7, *n* = 10; data reported as mean ± SEM). **(B)** Histograms from animals representing the frequency of circulating human T cells at weeks 2, 3, 4, and 5. **(C)** Percent change in humanized mouse body weight from the time of initial PBMC engraftment (*n* = 50); vertical blue line indicates the timepoint prior to the development of overt GvHD, red line denotes mean change in animal weight. **(D)** Parallel changes in body weight vs. relative proteinuria. **(E)** PBMC donor-dependent difference in the reduction of body weight post-PBMC engraftment.

The parental NSG mouse strain injected with human PBMCs attains adequate levels of peripheral T-cell engraftment for treatment studies between 3 and 4 weeks post-engraftment (21); however, between 4 and 5 weeks post-engraftment, severe GvHD develops (22). The PBMC humanized NSG model thus provides only a 1–2-week window in which immunotherapeutic modulation can be investigated (23–26). To expand the window of investigation, we utilized here the NSG-β2m^−/−^ variant, which is deficient in MHC class I and exhibits delayed development of overt GvHD (4). NSG-β2m^−/−^ mice were injected i.p. with 1 ×10^7^ healthy donor PBMCs and bled weekly to determine the level of human PBMC engraftment. Two weeks post-engraftment, ~10% of total live circulating cells stained positive for human CD3 (Figure 1A), which then increased to ~30% between weeks 4 and 5 (Figure 1A). Representative histograms (Figure 1B) demonstrate increasing frequencies of human peripheral T cells from weeks 2 through 5 post-engraftment. Following engraftment, ~98% of human CD45^+^ cells in the periphery were CD3^+^ (Supplemental Figure 1A).

Weight loss is a hallmark manifestation of overt GvHD (27), which develops with similar kinetics to that of peripheral human T-cell expansion. In the PBMC humanized NSG (parental) model, for example, weight loss begins at 3–4 weeks post-engraftment reaching a 15% reduction in body weight between 4 and 5 weeks post-engraftment (22). In order to evaluate weight loss in PBMC humanized NSG-β2m^−/−^ animals, a large cohort (*n* = 50) of mice were injected with PMBCs derived from a single healthy donor, followed by weekly weight measurements. Up to 5 weeks post-injection, no weight loss was observed. Animals began to exhibit weight loss between weeks 5 and 6 post-engraftment, with ~15% weight loss observed by 8 weeks post-engraftment of human PBMCs (Figure 1C). Overt GvHD in PBMC humanized NSG-β2m^−/−^ animals is thus delayed in comparison to humanization of the parental NSG strain, providing an extended time to explore immunotherapeutic modulation and/or anti-tumor response. Concurrent with weight loss, proteinuria was detected at increasing levels beginning at day 31 post-PBMC engraftment (Figure 1D), suggesting the initiation of GvHD at this timepoint. The strikingly similar kinetics of proteinuria and weight loss may suggest an additional physiologic metric for identifying the development of GvHD in PBMC humanized murine models.

A hypothesis attempting to explain the variability observed in experiments conducted with humanized mouse models is the utilization of different donor PBMCs. Similar to cancer patient populations, each individual PBMC donor may respond differently to both the tumor and the treatment. Prior data in support of this hypothesis were generated with NSG (parental) mice (21); however, there is little data to support this hypothesis in the NSG-β2m^−/−^ model. We engrafted NSG-β2m^−/−^ mice with 1 ×10^7^ human PBMCs from eight different healthy donors and followed the development of GvHD by weight loss (Figure 1E). With the exception of mice receiving PBMCs from donor #4, which began to exhibit weight loss at 4 weeks post-engraftment, mice receiving PBMCs from all other donors began losing weight at or after 5 weeks post-engraftment. Based on these results, we conclude that week 5 post-engraftment is the ideal endpoint for our anti-tumor studies.

### Bintrafusp Alfa Has Anti-tumor Efficacy in a Humanized Bladder Tumor Model

To investigate the anti-tumor effect of bintrafusp alfa in the PBMC humanized NSG-β2m^−/−^ mouse model, we first utilized the HTB-1 cell line, which is an HLA-A2^+^ human bladder transitional cell carcinoma line that expresses high levels of PD-L1 (Supplemental Figure 1). The dose of bintrafusp alfa used in these studies was 500 μg per injection, which is a dose similar to the 20 mg/kg maximum tolerated dose (MTD) in humans, as determined in the phase I clinical trial (28).

**Figure 2:**
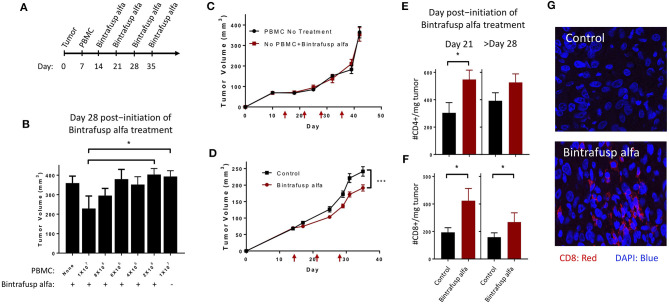
Bintrafusp alfa treatment results in anti-tumor efficacy in the HTB-1 subcutaneous bladder model. **(A)** Timeline of tumor injection, human PBMC engraftment, and bintrafusp alfa treatments. **(B)** Titration of the number of human PBMCs and their effect on bintrafusp alfa-mediated anti-tumor efficacy on day 28 post-initiation of bintrafusp alfa treatment (*n* > 4; *p < 0.05, student's *t*-test, error bars represent mean ± SEM). **(C)** Average growth curves of HTB-1 s.c. tumors in animals treated with bintrafusp alfa in the absence of human PBMCs (red) or in the presence of human PBMCs without bintrafusp alfa (black) (*n* ≥ 7). **(D)** Average growth curves of HTB-1 s.c. tumors in control-Ig and bintrafusp alfa-treated mice (data combined from 3 independent experiments, *n* ≥ 26, 2-way ANOVA, ***p < 0.0001; red arrows indicate bintrafusp alfa administration). HTB-1 tumor T-cell infiltrates for both CD4^+^ **(E)** and CD8^+^ **(F)** T cells per milligram tumor weight at day 21 (left, *n* = 9), or after day 28 (right, *n* = 22) post-initiation of bintrafusp alfa treatment. Error bars represent mean ± SEM; *p < 0.05. **(G)** Representative immunofluorescent images at day 28 post-initiation of bintrafusp alfa treatment, comparing CD8^+^ expression (red) in HTB-1 tumors from IgG control vs. bintrafusp alfa-treated mice.

Initially, we investigated if the magnitude of any anti-tumor effect mediated by bintrafusp alfa treatment in this model would be dependent on the initial number of injected human PBMC. To conduct these experiments, PBMC derived from a single donor (HLA-A2^+^) were injected in mice bearing HTB-1 tumors followed by weekly injections of bintrafusp alfa as indicated in Figure 2A. As shown in Figure 2B, injection of 1 ×10^7^ PBMCs followed by weekly injections of bintrafusp alfa elicited the most robust anti-tumor response. Half of this anti-tumor effect was eliminated by decreasing the initial number of injected PBMC to 8 ×10^6^ cells, with no anti-tumor effect observed when the initial number of injected PBMCs was reduced to 6 ×10^6^ cells and below (Figure 2B). To avoid experimental bias in subsequent studies, all animals injected with PBMC from a particular healthy donor were divided equally between treatment groups prior to the initiation of treatment.

Tumor growth kinetics of bintrafusp alfa-treated animals not receiving PBMCs was nearly identical to that observed in animals engrafted with 1 ×10^7^ PBMCs without bintrafusp alfa treatment, thus corroborating the need for both human PBMCs and bintrafusp alfa treatment for the observed anti-tumor effect (Figure 2C). In addition, the use of anti-PD-L1 (avelumab) showed no anti-tumor effect in this model (Supplemental Figure 1C).

For all remaining studies, animals were injected i.p. with 1 ×10^7^ human PBMCs; three independent anti-tumor studies were first conducted using three different PBMC donors (Figure 2D). The kinetics of the anti-tumor response was similar in each study with trending anti-tumor effect after day 7 post-initiation of bintrafusp alfa treatment (Figure 2D), and a significant anti-tumor response starting on day 14 post-initiation of bintrafusp alfa treatment, which remained significant until the end of study (> day 28 post-initiation of bintrafusp alfa treatment). Similar experiments were conducted with four additional PBMC donors (total of seven) in mice bearing HTB-1 tumors. Table 1 reports the average tumor weight at the end of study for mice in the control vs. bintrafusp alfa treatment groups, with the percent reduction of tumor weight induced by bintrafusp alfa treatment shown in the bottom row (bold numbers). As shown, bintrafusp alfa administration reduced tumor weight in mice engrafted with PBMCs from 7/7 donors, with a mean tumor weight reduction of 34.9%, ranging from 18 to 43% reduction. These data highlight the varied anti-tumor response that can be observed among mice engrafted with different PBMC donors.

**Table 1:** PBMC donor-dependent differences in the bintrafusp alfa-mediated anti-tumor response.

	**HTB-1**							**SiHa**		**MDA-MB-231**	
**PBMC donor**	**1**	**2**	**3**	**4**	**5**	**6**	**7**	**8**	**9**	**10**	**11**
Control tumor weight (mg)	250.71	342.42	393.20	440.39	250.71	231.57	228.25	276.14	472.11	197.73	363.03
Bintrafusp alfa tumor weight (mg)	164.52	239.05	228.89	250.21	164.52	189.71	131.57	16.86	328.88	225.93	215.22
% difference (Control/bintrafusp alfa)	**−34.38**	**−30.19**	**−41.79**	**−43.19**	**−34.38**	**−18.08**	**−42.36**	**−93.89**	**−30.34**	**14.26**	**−40.72**

*Bold values indicate bintrafusp alfa treatment*.

To better characterize the kinetics of the bintrafusp alfa-mediated anti-tumor response, we performed a longitudinal investigation of T-cell infiltration into the TME at days 7, 21, and >28 post-initiation of bintrafusp alfa treatment. At day 7 post-initiation of bintrafusp alfa treatment and prior to observing any anti-tumor effect, there was no difference in tumor infiltration with CD4^+^ or CD8^+^ T cells. Concomitant with a significant anti-tumor response at day 21 post-initiation of bintrafusp alfa treatment, there was an increased CD4^+^ (Figure 2E) and CD8^+^ (Figure 2F) T-cell presence per milligram tumor in bintrafusp alfa compared to control treated animals. Increased T-cell infiltration at day 21, prior to full engraftment of the peripheral T-cell compartment (Figure 1A), demonstrates the tumor-specific immune response conferred by bintrafusp alfa. At the end of study (> day 28 post-initiation of bintrafusp alfa treatment), there was a trend in the increase in CD4^+^ and a significant increase in CD8^+^ T-cell infiltration per milligram tumor when comparing bintrafusp alfa to control treated animals (Figures 2E,F). Enhanced CD8^+^ T-cell infiltration in bintrafusp alfa-treated vs. control tumors was also observed at day 28 post-initiation of bintrafusp alfa treatment via immunofluorescent analysis (Figure 2G).

### Bintrafusp Alfa Induces T-Cell Phenotypic Changes Within the Tumor Microenvironment

**Figure 3:**
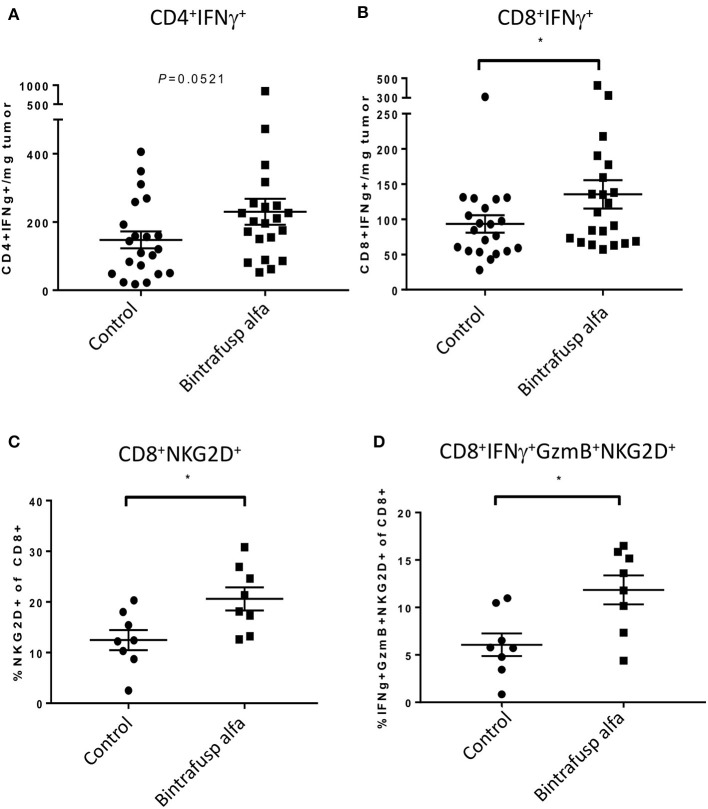
Bintrafusp alfa treatment is associated with increased T-cell infiltration/activation in the TME. Mice harboring HTB-1 subcutaneous tumors were treated with bintrafusp alfa or an IgG1 isotype control as outlined in Figure 2A. Number of IFNγ-producing CD4^+^ **(A)** and CD8^+^ **(B)** T cells per milligram tumor weight and frequency of **(C)** CD8^+^ NKG2D^+^ T cells and **(D)** IFNγ/GzmB^+^/NKG2D^+^ of total CD8^+^ tumor infiltrating cells at day 21 post-initiation of bintrafusp alfa treatment (*p < 0.05, student's *t*-test).

The level of tumor infiltrating human T cells expressing intracellular IFNγ was evaluated in HTB-1 tumors. At day 21 post-initiation of bintrafusp alfa treatment, a trending higher number of CD4^+^/IFNγ^+^ T cells (*p* = 0.0521) and a significant increase in CD8^+^/IFNγ^+^ T cells were observed in bintrafusp alfa-treated vs. control tumors (Figures 3A,B). In addition, CD8^+^ T cells also exhibited a marked increase in NKG2D expression (Figure 3C), suggesting a more active and innate phenotype (29, 30). Consistent with a more pro-inflammatory TME, a higher frequency of tumor-infiltrating CD8^+^ T cells co-expressing NKG2D, Granzyme B, and IFNγ was observed in bintrafusp alfa-treated animals (Figure 3D).

### Bintrafusp Alfa Treatment Results in Peripheral Sequestration of TGF-β

**Figure 4:**
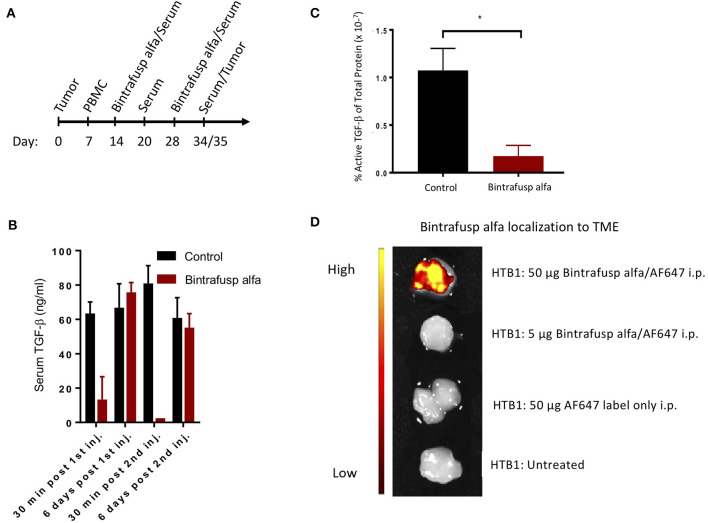
Robust peripheral and intratumoral changes associated with the administration of bintrafusp alfa in the humanized HTB-1 subcutaneous bladder model. **(A)** Timeline of PBMC engraftment, blood collection, and treatment for subcutaneous HTB-1 tumor-bearing PBMC humanized NSG-β2m^−/−^ mice. **(B)** Total serum TGF-β1 levels at 30 min and 6 days post first and second bintrafusp alfa injection (*n* = 6). **(C)** Active TGF-β1 levels as a percent of total protein in HTB-1 tumor lysates from control-IgG1 or bintrafusp alfa-treated mice at day 21 post-initiation of bintrafusp alfa treatment (*n* = 4; *p < 0.05). **(D)** *In vivo* localization of AF647-labeled bintrafusp alfa to HTB-1 tumors 2 weeks post i.p. injection.

The ability of bintrafusp alfa to sequester TGF-β from circulation as well as the TME was investigated using the treatment schedule described in Figure 4A. Levels of circulating TGF-β1 were measured via an ELISA assay. Despite elevated levels of TGF-β1 present in the serum of control, untreated mice, bintrafusp alfa effectively sequestered TGF-β1 to near the limits of detection within 30 min post-administration, with TGF-β1 returning to pre-bintrafusp alfa levels within 6 days (Figure 4B). One week after the last dose of bintrafusp alfa, tumors were homogenized, and a protein lysate was prepared for evaluation of active TGF-β1 via ELISA. We observed a >6-fold reduction in the amount of active TGF-β1 in the tumor following bintrafusp alfa treatment (Figure 4C). Consistent with this observation, there was a trending reduction in phopho-SMAD2 protein in the TME (Supplemental Figure 2).

### Bintrafusp Alfa Localizes and Modifies the Tumor Microenvironment

The PD-L1 IgG1 arm of bintrafusp alfa is designed to localize this therapeutic agent to the TME; HTB-1 tumors have previously been shown to express high levels of PD-L1 *in vivo* (19). To investigate the ability of bintrafusp alfa to localize to the TME, mice bearing HTB-1 tumors were injected with AlexaFluor 647 labeled bintrafusp alfa at a dose of 5 or 50 μg. To compensate for the relatively high levels of autofluorescence in the gastrointestinal tract, tumors were excised at 2 weeks post-injection of labeled bintrafusp alfa and imaged *ex vivo*. As shown in Figure 4D, tumors of animals receiving the higher dose of labeled bintrafusp alfa exhibited a positive fluorescent signal. This dose is 10% of the treatment dose. Additional organs were visualized, including heart, liver, brain, and reproductive tract; no visible off target accumulation was observed at this timepoint.

### Bintrafusp Alfa-Mediated Anti-tumor Response in HPV^+^ Cervical and Triple Negative Breast Cancer Models in PBMC Humanized Mice

**Figure 5:**
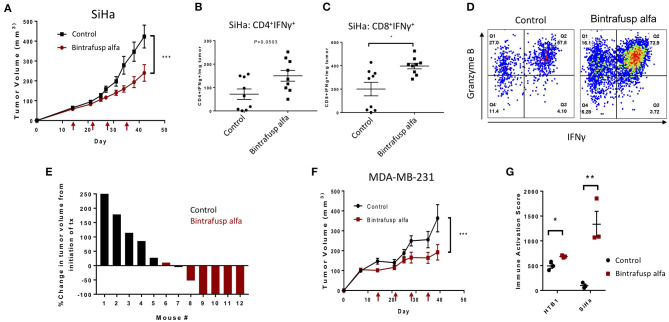
Bintrafusp alfa confers a robust anti-tumor effect in the HPV^+^ SiHa cervical and MDA-MB-231 triple negative breast cancer models. **(A)** Average SiHa tumor volumes from control (black) or bintrafusp alfa-treated (red) PBMC humanized mice (*n* = 21, 2-tailed ANOVA, ***p < 0.0001, data combined from 2 independent experiments). **(B,C)** Tumor infiltrating CD4^+^ **(B)** and CD8^+^ **(C)** IFNγ-producing T cells per milligram tumor weight at day 21 post-initiation of bintrafusp alfa treatment (*n* = 9, *p < 0.05, student's *t*-test). **(D)** Representative dot plots from the tumor of control (left) and bintrafusp alfa-treated (right) mice bearing SiHa tumors. IFNγ is represented on the x-axis and granzyme B on the y-axis. **(E)** Waterfall plot of the percent change in SiHa tumor volume from the initiation of treatment in one independent experiment at day 28 post-initiation of bintrafusp alfa treatment. **(F)** Average MDA-MB-231 tumor volumes from control-IgG1 (black) or bintrafusp alfa-treated (red) PBMC humanized mice (*n* = 9, 2-tailed ANOVA, ***p < 0.0001, data combined from 2 independent experiments). **(G)** Immune activation score derived from gene expression profiles (as described in Figure 6) from HTB-1 and SiHa tumors from control and bintrafusp alfa-treated mice (*n* = 3, *p < 0.05, **p < 0.01, student's *t*-test).

HPV positive HLA-A24^+^ cervical cancer cells (SiHa) were injected subcutaneously into the right flank of recipient animals and injections of HLA-A24^+^ PBMC and bintrafusp alfa were completed according to the previously described schedule (Figure 2A). A trending anti-tumor effect was observed after the second injection of bintrafusp alfa and a significant difference in tumor growth was observed after the third injection (Figure 5A). Consistent with an increased Th1 response, a trending increase in CD4^+^/IFNγ^+^ T cells (Figure 5B) and a significant increase in CD8^+^/IFNγ^+^ T cells (Figure 5C) per milligram tumor weight were observed in bintrafusp alfa-treated animals compared to controls. Additionally, multifunctional CD8^+^ T cells expressing both IFNγ and Granzyme B were observed in the TME of bintrafusp alfa-treated mice (Figure 5D). The magnitude of the bintrafusp alfa-mediated antitumor response in the SiHa model was dependent on PBMC donor (Table 1). The anti-tumor response ranged from a 30% reduction in tumor weight to near complete clearance with a different PBMC donor, where four of six treated animals harbored no detectable tumor (Figure 5E).

The bintrafusp alfa-mediated anti-tumor response was also investigated in the MDA-MB-231 triple negative breast cancer model. A varied anti-tumor response, which was dependent on PBMC donor, was again observed (Table 1). One PBMC donor (*n* = 6 mice) resulted in a >40% reduction in tumor volume, whereas the second PBMC donor (*n* = 3 mice) conferred no bintrafusp alfa-mediated anti-tumor response. The combination of these two independent experiments still resulted in an overall significant reduction in MDA-MB-231 tumor volume (Figure 5F) in response to bintrafusp alfa. The results of the immune activation score for each tumor type is shown in Figure 5G and will be detailed below.

### The TME Gene Signature Post-bintrafusp Alfa in the HTB-1 and SiHa Tumor Models

**Figure 6:**
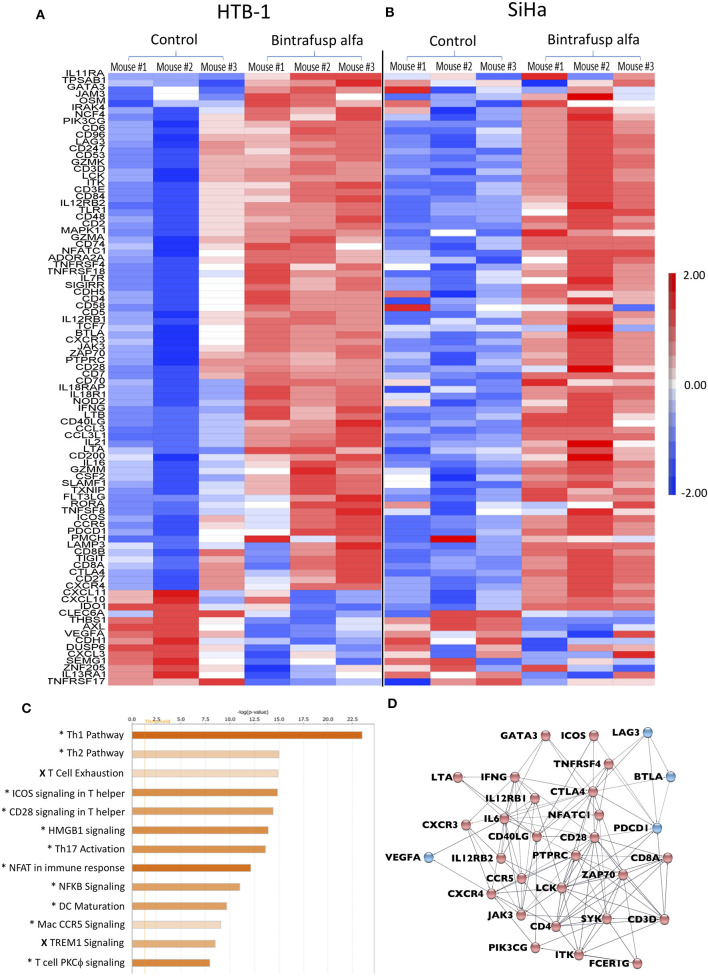
Distinct transcriptional changes within the TME following bintrafusp alfa treatment. **(A,B)** Heatmap RNA expression profile from a NanoString pan-cancer immune profiling panel assayed on enriched CD45^+^ cells from tumors of control and bintrafusp alfa-treated mice bearing HTB-1 tumors at day 21 post-initiation of bintrafusp alfa treatment **(A)** or SiHa tumors at day 22 post-initiation of bintrafusp alfa treatment **(B)** (fold change >1.5, ANOVA *p* < 0.1). **(C)** Ingenuity pathway analysis of HTB-1 and SiHa RNA expression profiles describing those pathways most altered with bintrafusp alfa treatment; (*) denotes pathways altered in both HTB-1 and SiHa tumors; (X) denotes pathways altered in SiHa tumors only. **(D)** String analysis of those genes altered in the top 5 pathways identified by Ingenuity Pathway Analysis. Red nodes denote those genes/pathways identified post-bintrafusp alfa treatment in both HTB-1 and SiHa tumors; blue nodes denote those genes/pathways identified post-bintrafusp alfa treatment in SiHa tumors only.

Using the described treatment schedule (Figure 2A), RNA was isolated from enriched human CD45^+^ cells from HTB-1 and SiHa tumors in bintrafusp alfa-treated and control animals at day 21 post-initiation of bintrafusp alfa treatment. Gene expression was evaluated via the NanoString pan-cancer immune profiling panel. Gene signature heatmaps corresponding to HTB-1 and SiHa control and bintrafusp alfa-treated tumors are shown in Figures 6A,B. In agreement with the results of the flow cytometry analysis of the TME, increased levels of CD4, CD8, CD3, and IFNγ mRNA expression were noted in both tumor types following bintrafusp alfa treatment, and a similar gene signature in response to bintrafusp alfa treatment was observed in both tumor models. However, differences in gene expression profiles among animals bearing the same tumor type were also noted after bintrafusp alfa treatment (Figure 6A).

Ingenuity pathway analysis (IPA) revealed conserved immune pathways altered in both tumor models (Figure 6C). Consistent with the results of the flow cytometry analysis of the TME (Figures 3A,B, 5B,C), the top altered IPA pathway revealed strong Th1 polarization of the immune response. Additional pathways involved in T-cell activation were also identified via IPA analysis, including ICOS, CD28, NFAT, NFκB, and PKCθ signaling. Two pathways identified via IPA analysis that were only upregulated in SiHa tumors, albeit to a lesser extent, were T-cell exhaustion and TREM1 signaling.

Genes comprising the top five identified IPA pathways are presented in a STRING analysis (Figure 6D), which serves to better visualize possible interactions and to better highlight those upregulated genes involved in immune activation after bintrafusp alfa treatment in both HTB-1 and SiHa tumors (red dots) and in SiHa tumors only (blue dots). Changes are seen in genes such as GATA3, LAG3, ICOS, CTLA4, IFNγ, CD28, ZAP70, LCK, CD4, and CD8. The STRING analysis demonstrates a network of genes consistent with increased T-cell infiltration and activation. Gene signatures consistent with immune migration include altered chemokine receptor expression such as CXCR3, CXCR4, and CCR5. Increased transcripts of CD3, CD4, and CD8 are consistent with increased T-cell presence within the TME. Those genes associated with altered TCR signaling include: ZAP70, LCK, JAK3, GATA3, SYK, and PTPRC. Additionally, transcripts associated with T-cell activation pathways are also modulated, including: CTLA-4, PDCD1, ICOS, and LAG3. Taken together, these gene expression data demonstrate a robust bintrafusp alfa-mediated T-cell centric immune signature within the TME translating to reduced tumor burden. Stewart et al. (20) have described an immune activation score that utilizes expression levels of T-cell-associated genes, immune activation markers, and MHC class II. The immune activation score measures levels of immune infiltration and activation and was demonstrated to clinically correlate with patient outcomes. Moreover, the immune activation score was similar to observations obtained using standard histological methods. Altered gene expression in the TME of both the SiHa and HTB-1 tumor models was quantitated via the immune activation score as described in the Materials and Methods (Figure 5G). A higher immune activation score was observed in both tumor models with bintrafusp alfa treatment.

### Reduced PD-1 Expression in CD8^+^ T Cells Is Associated With Bintrafusp Alfa Anti-tumor Responses

**Figure 7:**
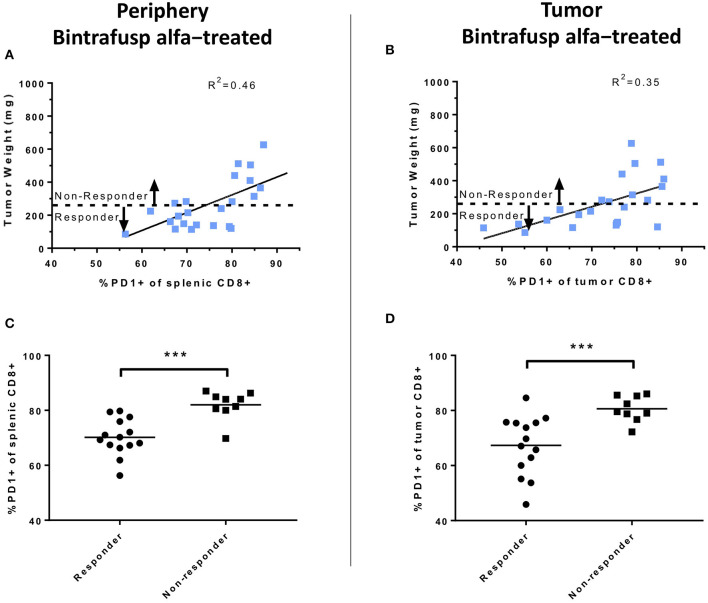
Analyses of peripheral and intratumoral CD8^+^ T-cell PD1 expression following bintrafusp alfa administration. **(A)** Association between PD1 expression in splenic **(A)** or intratumoral **(B)** CD8^+^ T cells vs. tumor weight at day 21 post-initiation of bintrafusp alfa treatment in bintrafusp alfa-treated PBMC humanized mice. **(C)** Splenic or **(D)** tumor CD8^+^ PD1 expression in animals responding to bintrafusp alfa (tumor weight <263 mg) vs. those not responding (tumor weight >263 mg) in HTB-1 humanized mice (***p < 0.0001, student's *t*-test).

The potential association between anti-tumor responses and expression of PD-1 on the surface of CD8^+^ T cells was also investigated. At day 21 post-initiation of bintrafusp alfa treatment, there was a linear relationship between the percentage of PD-1^+^ CD8^+^ T cells in the spleen and the tumor mass in bintrafusp alfa-treated animals (linear regression, R^2^ = 0.46; Figure 7A). A similar linear relationship was observed between tumor mass and PD-1 expression in tumor-infiltrating CD8^+^ T cells in bintrafusp alfa-treated animals (linear regression, R^2^ = 0.35; Figure 7B). Those animals with a tumor mass lower than the average of bintrafusp alfa-treated animals were noted as “Responders,” whereas those animals with a tumor mass higher than the average were indicated as “Non-responders.” As shown in Figures 7C,D, the percentage of PD-1 expression in peripheral and tumor-infiltrating CD8^+^ T cells, respectively, was significantly lower in responders to bintrafusp alfa when compared with non-responders. The implications for this finding will be discussed below.

## Discussion

While no animal model can serve as an absolute surrogate for clinical studies, different animal models, each with its own strengths and weaknesses, can provide valuable information in understanding a novel immunotherapeutic agent. Many immunotherapeutic agents are human proteins and thus repeated administrations will inevitably lead to xenogeneic anti-agent murine immune responses. In addition, human cytokines are often species-specific in terms of activity, toxicity or potency. The NSG model affords the evaluation of immunotherapeutic agents employing both human immune cells and human tumor cells. There are several humanized mouse models [reviewed in De La Rochere et al. (31)] made up of a constellation of variables and components, including source of immune cells, quantity of immune cells, timing of engraftment, and mouse line. Early work with SCID (32) and athymic nude mice (33) revealed the ability of immunodeficient lines to accept human xenografts; however, due to remaining murine immune populations, these animals were inefficient at harboring human immune cells. Development of the NSG, NOG, and NRG lines allowed for more tolerant engraftment of human immune cells due to the lack of a murine adaptive immune response and a defective innate immune response. These lines similarly share the ability to accept and harbor human PBMCs, making them interchangeable for many avenues of investigation. While initially tolerant of human PBMCs, these lines rapidly develop GvHD due to a human PBMC-generated xenogeneic response. Several genetic variants eliminating murine MHC class I and/or II were created to delay or prevent the development of GvHD. The NSG-β2m^−/−^ mouse was previously reported to exhibit delayed development of GvHD due to the lack of beta-2 microglobulin, a component of MHC class I, which was also seen in the studies reported here. Additionally, the NSG-β2m^−/−^ variant also lacks FcRn, which results in reduced antibody stability (1); while this should be a concern for any study investigating an antibody-based immunotherapy, the current study demonstrates efficacy despite the lack of FcRn. Similarly, the NSG K^d−/−^D^b−/−^ line was created (34) that lacks murine MHC class I while maintaining FcRn. More recently, the NSG IA^−/−^K^d−/−^D^b−/−^ line was created that lacks both murine MHC class I and II (1). An additional MHC class I/II knockout mouse was also created, the NOG dKO line, which is deficient in β2m (5). Both lines show further delay of GvHD. Additional investigation may show these two lines to be more suited for accepting human PBMCs.

In the present study we have employed the human bifunctional immunotherapy agent anti-PD-L1/TGFβRII (bintrafusp alfa) to evaluate both the anti-PD-L1 activity and TGFβ “trapping” of this molecule. Only 3–4 weekly injections of bintrafusp alfa were given, starting 1-week post-human PBMC engraftment, due to the onset of GvHD. PBMCs from multiple donors were evaluated using tumors from three different human carcinoma cell types. In an effort to conserve human PBMCs, the number of PBMCs engrafted per mouse was titrated and it was determined that 1 ×10^7^ PBMCs/mouse/injection gave optimal anti-tumor effects with the use of bintrafusp alfa. As shown, both donor PBMCs and bintrafusp alfa were needed for anti-tumor activity (Figures 2B,C). When first analyzed at day 7 post-initiation of bintrafusp alfa, equal numbers of both human CD4^+^ and CD8^+^ T cells were observed in tumors of control and bintrafusp alfa-treated mice; however, at day 21 and > day 28 post-initiation of bintrafusp alfa treatment, there were clear differences in CD4^+^ and CD8^+^ tumor-infiltrating lymphocytes (TILs) in bintrafusp alfa-treated mice vs. control. Activation status of both CD4^+^ and CD8^+^ TILs was seen in their expression of IFNγ and NKG2D. We observed a robust cellular response to bintrafusp alfa within the TME, which is characterized by increased T-cell infiltration (Figures 2E–G) and activation (Figures 3A–D). Increased CD8^+^ T-cell infiltration into the TME as determined via flow cytometry (Figure 2F) was confirmed via fluorescent microscopy (Figure 2G) and gene expression analysis (Figure 6). Increased T-cell activation determined via flow cytometry (Figures 3, 5B,C) was further confirmed and characterized via gene expression analysis (Figures 5G, 6). There was also a conserved phenotypic change within the TME for both HTB-1 and SiHa tumors. Detailed analysis of gene expression within the TME may help to identify additional targets to be explored in combination with bintrafusp alfa in future studies. For example, upregulation of IL-12r or CTLA-4 (Figure 6) in both the HTB-1 and SiHa models may suggest a combination with an IL-12 or CTLA-4 modulating agent which may enhance the observed anti-tumor response.

Bintrafusp alfa is shown here to localize to the tumor as well as to reduce TGF-β1 in the TME and in the periphery, exemplifying the bifunctional nature of this molecule. It should be pointed out that while TGF-β1 levels returned to normal in the periphery 6 days following bintrafusp alfa administration, active TGF-β1 was still significantly reduced in the TME, thus providing evidence that bintrafusp alfa may be more effective at trapping TGF-β in the TME than in the periphery. When compared to control mice, bintrafusp alfa-treated mice showed no differences in the levels of CD4^+^ or CD8^+^ T cells in the periphery, but higher levels were observed in the TME of bintrafusp alfa-treated tumors. There was also a reduction in the level of PD1^+^ CD8^+^ T cells in both the TME and in the periphery in mice that exhibited anti-tumor effects with bintrafusp alfa treatment. Based on these results, we postulate that reduction of PD1^+^ CD8^+^ T cells in the periphery could be evaluated as one possible correlate of clinical response in bintrafusp alfa-treated patients. In addition, future studies could include analyses of antigen spreading, anti-idiotype antibody development, TCR clonality and single cell RNAseq in the TME.

In addition to the bladder carcinoma model, anti-tumor efficacy and immune effects were seen using bintrafusp alfa in the HPV^+^ SiHa cervical carcinoma and the MDA-MB-231 triple negative breast carcinoma models. Gene expression arrays of the CD45^+^ cells in the TME of HTB-1 and SiHa tumors in bintrafusp alfa-treated mice, moreover, clearly show T-cell activation profiles with enhancement of the Th1 pathways in both tumor types as well as an increase in the immune activation score (Figures 5G, 6). Of note in these studies is the variance that can exist in the use of different apparently healthy donors in terms of anti-tumor effect with a given immunotherapeutic agent. This was observed in each of the three carcinoma models employed (Table 1). The donor dependent variability in anti-tumor response is shown to emulate the varied clinical response in patient populations. While there was a ranging degree of response, the ability of bintrafusp alfa to reduce tumor burden was conserved using PBMC from most donors (10/11 donors, Table 1). Examples of these differences are shown in Supplemental Figure 3. While many determinants likely contribute to therapeutic response, a correlation between tumor mass and both peripheral and intratumoral CD8^+^ PD1 expression was observed in those animals exhibiting the most robust therapy-mediated anti-tumor response (Figure 7). Future studies may help to elucidate additional prognostic components to the therapeutic anti-tumor response. Varied control tumor growth in mice humanized from a single PBMC donor could be due to the specific tumor line used. For instance, little variability is observed in control animals harboring HTB-1 tumors (Figure 2D), whereas those animals engrafted with either SiHa (Figures 5A,E) or MDA-MB-231 (Figure 5F) cells exhibit more variability in tumor growth. This variability is more evident when defining tumor growth as percent change in initial tumor volume (Figure 5E), compared with monitoring tumor growth rates (Figure 5A). Despite observed variability, it remained that tumor growth control was attained in animals administered bintrafusp alfa.

The phase I clinical study (15) of bintrafusp alfa did not include tumor biopsies and thus no comparisons could be made concerning immune cell changes in the TME vs. the periphery. There have been two prior comprehensive reports of bintrafusp alfa in syngeneic murine models (9, 11); however, this is the first report of changes in the gene signature of human immune cells in the TME induced by bintrafusp alfa in human xenograft models.

As described above, donor PBMC titration studies were carried out to determine the minimal number of PBMCs to be engrafted to obtain a maximal anti-tumor effect. Conservation of PBMCs will be of importance in the use of cancer patients' PBMCs both prior to, and at multiple time points, post-administration of a given immunotherapeutic agent or other type of therapy.

While several previous studies from our group and others have investigated in detail the activity of bintrafusp alfa *in vitro* and *in vivo* in syngeneic murine models of cancer, the present study investigates for the first time the activity of this bifunctional agent against human xenografts in the context of human immune cells in humanized mice. Utilization of humanized mice not only allowed for the investigation of bintrafusp alfa's effect on human T cells *in vivo* and their pattern of tumor infiltration following administration of the agent, but also permitted repeated administrations of the agent over several weeks, which has not been possible with syngeneic mice due to xenogeneic reaction against this human reagent. Currently, there are multiple clinical studies investigating the use of bintrafusp alfa in combination with other immunotherapeutics, including an actively accruing phase I/II clinical trial (NCT03493945) evaluating a brachyury-targeted antitumor vaccine, bintrafusp alfa, an IL-15 agonist (N-803), and an IDO1 inhibitor in metastatic castration-resistant prostate cancer (35). Preclinical evaluation of such combinations involving the use of several human reagents will be facilitated using a humanized murine model system as the one described here. We thus believe that the results of this study constitute the backbone for future investigations of combinations of bintrafusp alfa with other agents, including human or humanized biologicals, to form the rationale for future combinatorial clinical studies. For example, this model could be used to evaluate the response to bintrafusp alfa employing the PBMC from patients who will undergo bintrafusp alfa therapy to see if there is a correlation between the anti-tumor results in this model and clinical benefit. In addition, one can compare the PBMC pre- and post-bintrafusp alfa in this model with the use of an additional immunotherapeutic to help determine the potential benefit that may be derived from the use of the additional agent.

## Data Availability Statement

The datasets generated for this study are available on request to the corresponding author.

## Ethics Statement

The animal study was reviewed and approved by the NIH Intramural Animal Care and Use Committee.

## Author Contributions

YM, JG, and CP conceived and designed the study. YM, AS, and JG developed the methodology. JS supervised the study. All authors contributed to the acquisition of data, analysis and interpretation of data, and writing of the manuscript.

## Conflict of Interest

The authors declare that the research was conducted in the absence of any commercial or financial relationships that could be construed as a potential conflict of interest.

## Abbreviations

β2m: beta-2 microglobulin
CRADA: Cooperative Research and Development Agreement
GvHD: Graft vs. Host Disease
HPV: human papilloma virus
IPA: ingenuity pathway analysis
MTD: maximum tolerated dose
ORR: overall response rate
PBMC: peripheral blood mononuclear cell
TCR: T-cell receptor
TME: tumor microenvironment.
