# Erratum: The Use of a Humanized NSG-β2m^−/−^ Model for Investigation of Immune and Anti-tumor Effects Mediated by the Bifunctional Immunotherapeutic Bintrafusp Alfa

**DOI:** 10.3389/fonc.2020.01548

**Published:** 2020-07-24

**Authors:** Frontiers Production Office

**Affiliations:** Frontiers Media SA, Lausanne, Switzerland

**Keywords:** humanized mouse, NSG-β2m^−/−^, bintrafusp alfa, immunotherapy, TGF-β

Due to a production error, an author name was incorrectly written as “Y. Maurice II Morillon.” The correct name is “Y. Maurice Morillon II.” The publisher apologizes for this mistake. The original article has been updated.

