# Supplementary material for: The Use of a Humanized NSG-β2m−/− Model for Investigation of Immune and Anti-tumor Effects Mediated by the Bifunctional Immunotherapeutic Bintrafusp Alfa
Source: Front Oncol. 2020 Apr 21;10:549. doi: 10.3389/fonc.2020.00549 (PMC7186351; doi:10.3389/fonc.2020.00549)
Supplement: Supplementary file 1 [file Image_1.PDF]

Supplemental Figure S1.

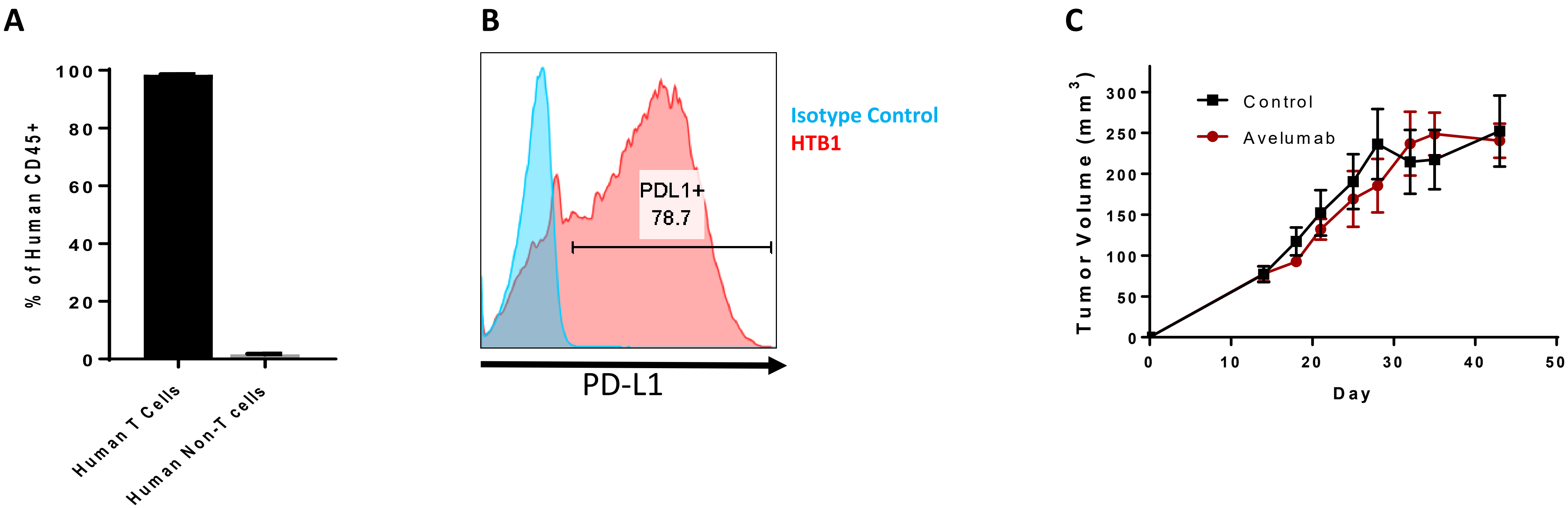

Supplemental Figure S1. (A) Engraftment of human immune cells into NSG-β2m$^{-/-}$ mice. NSG-β2m$^{-/-}$ mice were injected with 1X10^{7} human PBMC then bled 4 weeks post-engraftment. Immunostained cells were gated on live singlets. Shown is the frequency of human CD3^{+}/CD45^{+} T cells vs. CD3^{-}/CD45^{+} non-T cells, as determined via cytometric analysis. (B) HTB1 in vitro PD-L1 expression. Representative histogram of HTB1 cells immunostained for surface expression of PD-L1. (C) No anti-tumor effect conferred by anti-PD-L1 avelumab in the HTB1 PBMC humanized NSG-β2m$^{-/-}$ mouse model. PBMC humanized HTB1 tumor-bearing animals were administered 400 µg avelumab i.p. weekly (n=7).

Supplemental Figure S2.

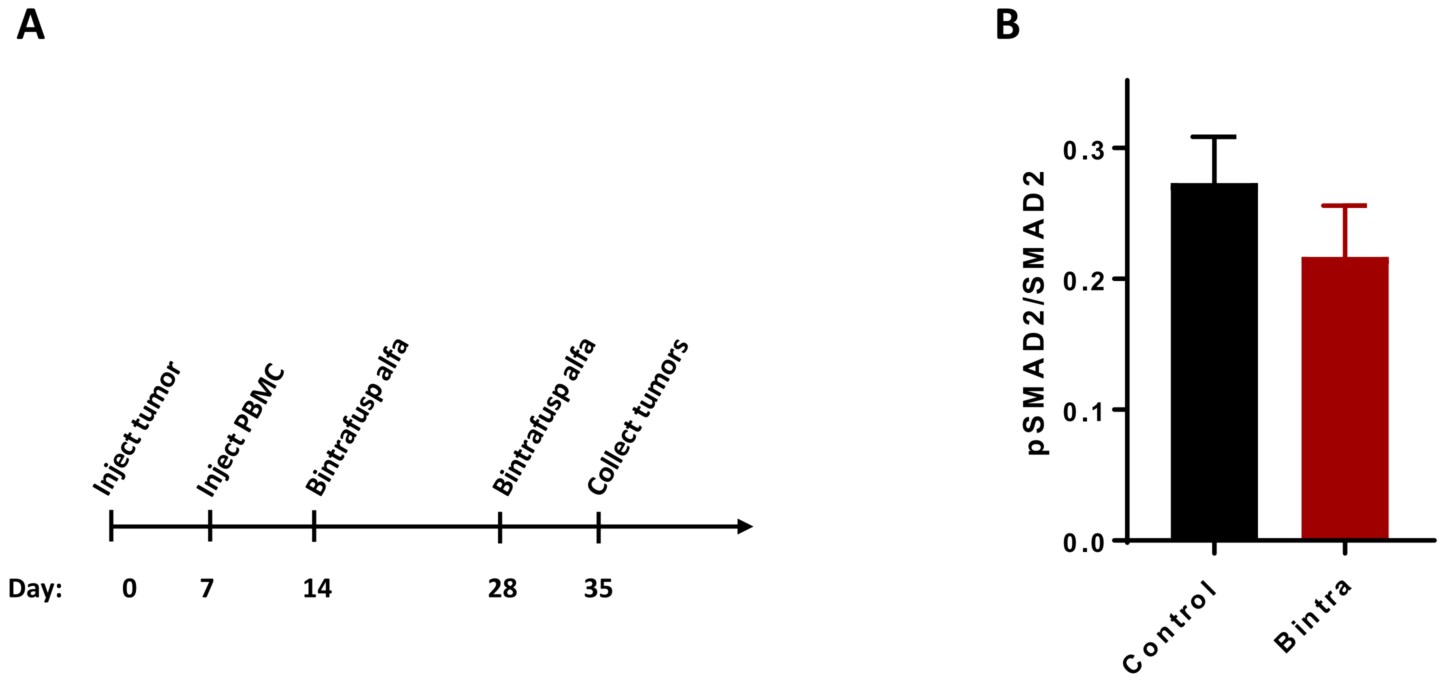

Supplemental Figure S2. Active TGF-β is sequestered in the tumors of bintrafusp alfa-treated animals, resulting in reduced phospho-SMAD2 within the TME. HTB-1 tumor bearing PBMC humanized animals were administered bintrafusp alfa as indicated in the schema above (A), followed by the collection of tumors 7 days after the last injection. Tumor lysates were evaluated for levels of pSMAD2 and total SMAD2 (B). Data reported as pSMAD2 normalized to total SMAD2 (n=3).

Supplemental Figure S3.

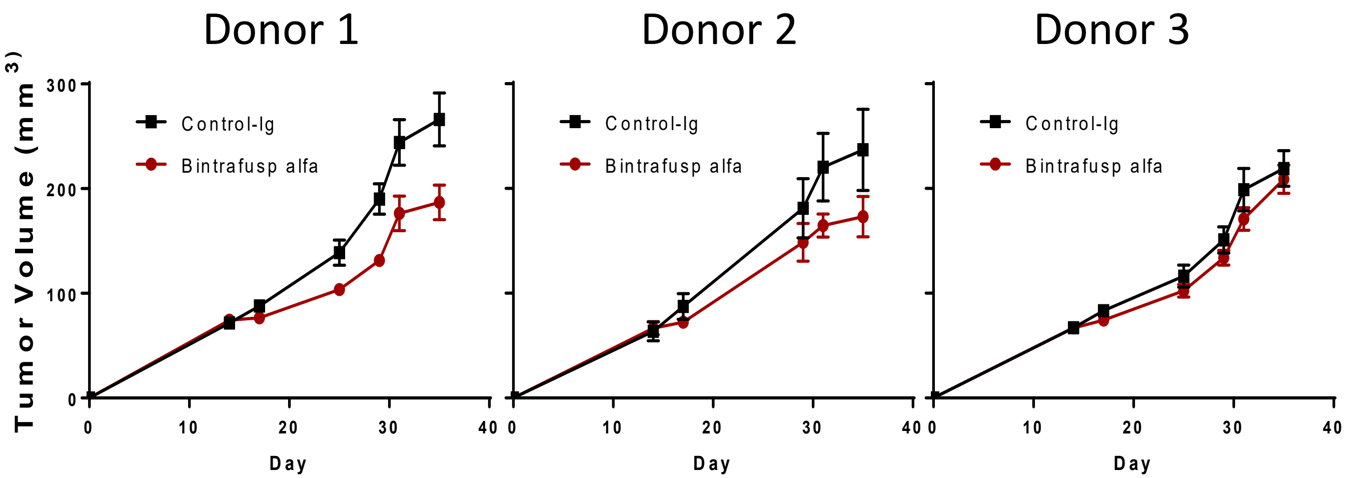

Supplemental Figure S3. Anti-tumor effect by PBMC donor following bintrafusp alfa treatment in the HTB1 PBMC humanized NSG-β2m$^{-/-}$ mouse model. Animals were injected with 5 x 10^6 HTB1 tumor cells followed by injection of 1 X 10^7 healthy donor PBMC. 500 µg bintrafusp alfa was injected i.p. at days 14, 21, and 28.
